# A Path-Driven Fluid Routing and Scheduling Method for Continuous-Flow Microfluidic Biochips with Delay Time Optimization

**DOI:** 10.3390/mi16060625

**Published:** 2025-05-26

**Authors:** Zhisheng Chen, Bowen Liu, Hongjin Su, Zhen Chen, Genggeng Liu, Xing Huang

**Affiliations:** 1 School of Informatics, Xiamen University, Xiamen 361004, China; czs23@xmu.edu.cn; 2 College of Computer and Data Science, Fuzhou University, Fuzhou 350116, China; 231010011@fzu.edu.cn (B.L.); 231020037@fzu.edu.cn (H.S.); liugenggeng@fzu.edu.cn (G.L.); 3 Engineering Research Center of Big Data Intelligence, Ministry of Education, Fuzhou University, Fuzhou 350116, China; 4 Fujian Key Laboratory of Network Computing and Intelligent Information Processing, Fuzhou University, Fuzhou 350116, China; 5 School of Big Data, Fuzhou University of International Studies and Trade, Fuzhou 350202, China; xing.huang1010@gmail.com

**Keywords:** continuous-flow microfluidic biochips, routing, application mapping, particle swarm optimization, conflict awareness

## Abstract

Routing and application mapping are critical stages in the design of continuous-flow microfluidic biochips (CFMBs). The routing stage determines the channel network connecting components and ports, while application mapping schedules fluid transportation and wash operations based on the designed biochip architecture. Existing methods typically handle these stages separately: routing focuses solely on physical metrics without considering subsequent scheduling requirements, while application mapping adopts one-shot scheduling strategies that can lead to suboptimal solutions. This paper proposes an integrated path-driven methodology that jointly optimizes routing and application mapping. For routing, we develop a hybrid particle swarm optimization algorithm that incorporates conflict awareness and channel utilization strategies. For application mapping, we introduce an iterative approach that leverages historical scheduling information to progressively optimize fluidic-handling and wash operations. Experimental results on both real and synthetic benchmarks demonstrate significant improvements over state-of-the-art methods, achieving reductions of 22.05% in total channel length, 21.79% in intersections, 21.97% in total delay time, and 8.30% in biochemical reaction completion time. The proposed methodology provides an effective solution for the automated design of CFMBs with enhanced physical and operational efficiency.

## 1. Introduction

Continuous-flow microfluidic biochips (CFMBs) have revolutionized biochemical analysis by miniaturizing traditional laboratory processes on a single chip [1,2]. By integrating various functional components such as mixers, filters, and detectors at the sub-millimeter scale, these devices enable processing samples in nanoliter volumes while maintaining high detection sensitivity [3,4]. The high degree of automation in CFMBs significantly accelerates experimental procedures, finding widespread applications in clinical diagnostics, DNA analysis, drug discovery, and environmental monitoring, among others [4,5,6,7,8,9]. The ability to perform complex biochemical protocols efficiently while reducing reagent consumption has established CFMBs as a promising platform for practical applications [10].

**Figure 1:**
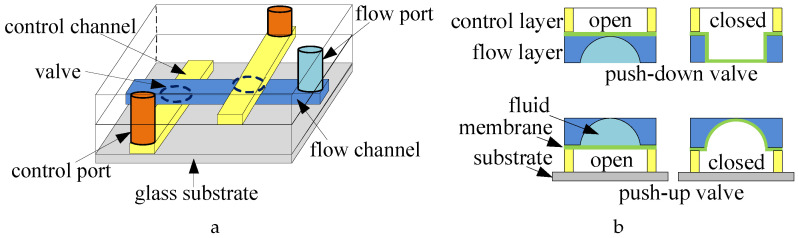
(**a**) Schematic of a three-layer biochip. (**b**) Push-down and push-up valves [11].

A typical CFMB consists of two layers: a flow layer and a control layer. The flow layer contains channels for fluid transportation, components for biochemical operations, and input/output ports for fluid injection and collection. The control layer contains pneumatic control channels and valves to regulate fluid flow. A flexible membrane that acts as a valve is formed at the overlapping area between channels of the two layers [3]. The structure of a CFMB is shown in Figure 1a, where the control layer can be placed both above or below the flow layer. The biochip is composed of a polydimethylsiloxane (PDMS) elastomer layer for channels and valves, bonded onto a glass substrate. When pressure is applied to the control channel, the membrane deforms: in the case of an upper-layer control channel, the membrane bends downward into the underlying flow channel; in the case of a lower-layer control channel, the membrane bends upward into the flow channel above. This deformation effectively seals the flow channel and prevents fluid from passing through. Upon release of the air pressure, the membrane returns to its original position, thereby creating a push-down (push-up) valve, as depicted in Figure 1b.

The flow-layer design of CFMBs involves two key stages: architectural synthesis and application mapping. Architectural synthesis can be further divided into high-level synthesis and physical design [12,13]. High-level synthesis determines the binding and scheduling of biochemical operations, generating the component interconnection requirements. Physical design then performs component placement and channel routing to create the detailed chip layout, aiming to minimize channel length, intersections, and chip area [14,15]. The application mapping stage, based on the physical architecture, determines the detailed scheduling of fluid transportation and wash operations [16,17]. This includes allocating fluid paths for each transportation task, scheduling wash operations to prevent cross-contamination, and coordinating multiple fluid flows to avoid channel conflicts. As biochemical applications grow in complexity, with protocols requiring thousands of valves to execute parallel operations [3], the manual design of CFMBs has become extremely challenging and time-consuming. Traditional manual methods can no longer handle the increasing design complexity, especially when considering multiple optimization objectives such as completion time, chip area, and manufacturing cost. This has led to growing interest in computer-aided design (CAD) methodologies for automated CFMB design. CAD tools can systematically explore the vast design space, balance multiple constraints, and generate optimized solutions more efficiently than manual approaches [18].

Channel routing and application mapping are two critical and closely interrelated problems in CFMB design. The routing stage is critical in determining the physical layout of microfluidic channels that connect components and ports for sample dispensing and waste collection. As the complexity of CFMBs grows, with designs featuring hundreds of individually addressable chambers, the routing challenges become increasingly significant. While key optimization objectives traditionally include minimizing total channel length and reducing intersections to control manufacturing costs, these physical metrics alone are insufficient for modern CFMB design [19,20]. Long flow channels not only increase vulnerability to physical defects such as leakage and blocking but also impact fluid quality due to extended transportation times [21]. This is particularly critical for time-sensitive applications like clinical diagnostics and point-of-care testing, where delayed fluid arrival can lead to unexpected errors. Furthermore, existing routing approaches primarily focus on physical metrics while overlooking the impact on subsequent fluid transportation scheduling [22,23]. When channels need to make detours to avoid blocked components, this not only increases the total channel length but also creates potential conflicts between fluid transportation tasks, ultimately extending biochemical reaction completion times. This suggests the need for more comprehensive routing strategies that consider both physical constraints and operational efficiency.

The application mapping stage assigns and schedules fluid transportation and wash operations to the routed channels based on the physical architecture. A major challenge is minimizing delays caused by channel conflicts, where multiple fluid transportation tasks compete for the same channel segments simultaneously [16]. These conflicts are particularly complex as flow paths often share multiple channel segments and intersections, making conflict-free scheduling crucial for operational efficiency. Current mapping methods typically adopt a one-shot scheduling approach, making greedy decisions without considering the global impact on system performance [16,19]. Additionally, wash operations to remove cross-contamination are often planned opportunistically without considering their impact on fluid transportation schedules. This uncoordinated scheduling of transportation and wash tasks can significantly extend the overall completion time of biochemical reactions.

Current CAD flows, however, treat physical routing and application mapping as decoupled optimization problems. Physical routers minimize geometric metrics such as total channel length and crossings, yet ignore the downstream scheduling conflicts that arise when multiple fluids concurrently share channel segments. Conversely, schedulers often rely on greedy, one-shot heuristics that assume fixed routing and inelastic wash strategies—an assumption that inflates latency and reagent consumption in complex assays. To close this gap, we propose a path-driven, conflict-aware framework that jointly optimizes routing and scheduling. By embedding congestion penalties into a hybrid particle-swarm router and by iteratively refining operation order and wash planning, our method realizes a co-design loop that lowers both manufacturing cost (shorter, cleaner channels) and assay latency. The main contributions are as follows.

- We develop a path-driven conflict-aware routing algorithm based on hybrid particle swarm optimization (DPSO). The algorithm starts with a conflict-aware initialization strategy to reduce connection pairs, followed by a preprocessing scheme that efficiently handles obstacle and crossing constraints. To enhance the search capability, we integrate orthogonal experimental design and genetic operators into the DPSO framework. The algorithm also incorporates path adjustment and refinement strategies to optimize channel utilization while considering potential transportation conflicts.
- We propose a path-driven iterative application mapping approach that breaks through the limitations of traditional one-shot scheduling. Unlike existing methods, our approach performs multiple rounds of scheduling to progressively optimize the solution. By leveraging historical scheduling information from previous iterations, the algorithm makes informed decisions about operation ordering. The approach also features a globally aware washing strategy and a flexible cleaning path adjustment mechanism, significantly reducing transportation task delays caused by wash operations.
- We conduct comprehensive experiments to evaluate our methodology using both real and synthetic benchmarks. The results demonstrate significant improvements over state-of-the-art methods across multiple metrics: a 22.05% reduction in total channel length, a 21.79% reduction in number of crossings, a 21.97% reduction in total delay time, and an 8.30% reduction in biochemical reaction completion time. These improvements validate the effectiveness of our integrated path-driven approach in optimizing both physical design metrics and operational efficiency.

The rest of the paper is organized as follows: Section 2 analyzes related works. Section 3 presents the problem formulation. Section 4 introduces the details of the proposed algorithm. Section 5 shows the experimental results. Section 6 discusses the future prospects. Conclusions are given in Section 7.

## 2. Related Work

### 2.1. Architectural Synthesis

In recent years, significant progress has been made in flow-layer architecture synthesis methods for CFMBs. The PathDriver method [20] pioneered the solution to air elimination problems in practical fluid transportation operations by constructing an integer linear programming (ILP) model to optimize flow paths. Building on this, the PathDriver architecture introduced X-structure routing and fluid port minimization strategies, which not only reduced the total length of flow channels and number of intersections but also significantly improved the reliability of biochips. In terms of routing algorithms, researchers have proposed various innovative methods. Reference [24] combined the genetic algorithm (GA) with the A* algorithm to construct an automatic routing framework. Reference [22] developed an efficient bi-criteria Steiner tree algorithm to simultaneously minimize the total wirelength and diameter, which corresponds to the total length and the longest length of flow channels in CFMBs, respectively. Reference [23] broke through traditional limitations by proposing an algorithm supporting arbitrary angle routing, achieving channel length optimization while maintaining routing flexibility. In the field of chip architecture optimization, a series of comprehensive methods have been proposed. Reference [25] achieved coordinated design of component placement and channel routing schemes based on sequence pair representation, combining simulated annealing algorithm and negotiated routing methods.

However, existing design methods focus too heavily on optimizing traditional metrics such as total channel length and the number of intersections, often neglecting potential conflicts that may occur when flow paths execute fluid transportation tasks. These conflicts not only affect chip operation efficiency but may also lead to significant increases in biochemical reaction completion times.

### 2.2. Application Mapping

After completing high-level synthesis and placement/routing, although preliminary binding, scheduling results, and biochip architecture can be obtained, these results still have certain limitations. Since fluid channel design has not been completed during the high-level synthesis phase, fluid transportation time must be simplified to a fixed constant, leading to the inaccurate estimation of biochemical reaction execution times. Therefore, it is necessary to precisely bind fluid transportation tasks, fluid storage tasks, and wash operations to corresponding channels and ports through application mapping, while binding operations to appropriate components, ultimately obtaining an exact scheduling scheme that satisfies all constraints.

In application mapping research, reference [26] proposed an innovative greedy resource binding algorithm that improves CFMBs’ reliability by minimizing valve switching to reduce fluid transportation tasks. However, when different fluids flow through the same channel or component sequentially, subsequent fluids may be contaminated by residual samples, potentially causing errors in biochemical reaction results. To address contamination issues, researchers have proposed a series of wash optimization methods. Reference [27] pioneered a washing optimization method that establishes a path dictionary by pre-searching feasible paths in the chip architecture and calculates optimal wash paths based on washing priority objectives to complete contaminated channel cleaning in minimal time. Reference [28] deepened the washing problem model by proposing a wash path calculation method supporting multiple contaminants. Reference [16] innovatively integrated washing and scheduling into application mapping, using a list scheduling heuristic method to automatically trigger wash operations when contamination is detected. In a comprehensive design, reference [19] proposed a top-down flow layer design method that integrates distributed storage into application mapping while coordinating high-level synthesis, placement/routing, and washing optimization issues. Reference [17] further proposed an application mapping-control co-design scheme for distributed channel storage microfluidic biochips, simultaneously addressing application mapping, valve synchronization, and control system design through a systematic synthesis process.

However, existing research has a common problem: effective information generated during scheduling is often ignored due to the lack of multi-round scheduling mechanisms. This forces fluid transportation and wash operations to rely on greedy strategies for scheduling, increasing the probability of task conflicts and ultimately extending biochemical reaction completion times. To address this issue, this paper innovatively proposes introducing a multi-round scheduling mechanism in the application mapping phase, fully utilizing information generated from each round of scheduling to obtain superior application mapping solutions.

To address the aforementioned limitations in existing work, our proposed methodology integrates a conflict-aware hybrid particle swarm optimization (HPSO-CAR) and a multi-round iterative scheduling algorithm. HPSO-CAR incorporates congestion time as a penalty term in the routing objective function to proactively minimize channel conflicts during the routing phase. Meanwhile, the iterative application mapping strategy breaks the limitations of greedy one-shot scheduling by leveraging historical scheduling information to refine operation ordering and wash planning. This joint approach ensures improved channel utilization, reduced wash frequency, and lower biochemical reaction completion time, effectively overcoming the deficiencies highlighted in prior methods.

## 3. Design Challenges and Problem Formulation

In this section, we first present the relevant concept and design challenge of the routing phase, followed by an explanation of the concept and design challenge in the application mapping phase, and finally, we present the problem model.

### 3.1. Basic Concept and Design Challenge of Fluid Routing

**Figure 2:**
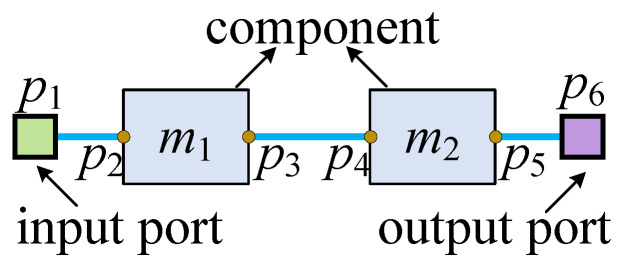
A complete fluid transportation path.

The integrated binding, scheduling, and placement results serve as the foundational input for the routing process. This process requires consideration of several key elements: connection pairs and their associated fluid transportation task execution time intervals, component coordinates, and their dimensions. In this framework, nodes are defined as flow ports or component interfaces (e.g., $p_{1}$–$p_{6}$, as shown in Figure 2). Each connection pair consists of a source node and a target node, with the primary task of routing being the establishment of appropriate connections between these nodes. Furthermore, connection pairs are assigned specific fluid transportation task time intervals, as detailed in Table 1.

A key constraint to be followed during the routing process is that channels must not pass through the interior of components. When two connection pairs (such as $Con_{1}$ and $Con_{2}$) share or cross channels, the overlapping portion of their fluid transportation task time intervals is defined as the conflict time. For example, in Table 1, connection pairs $Con_{2}$ and $Con_{3}$ have a conflict time of 6 s in the case of channel sharing or crossing.

A complete fluid transportation path typically consists of multiple connection pairs. Taking the fluid transportation from components $m_{1}$ to $m_{2}$ shown in Figure 2 as an example, the path consists of three connection pairs: $p_{1}$ (input port)–$p_{2}$ (component interface), $p_{3}$ (component interface)–$p_{4}$ (component interface), and $p_{5}$ (component interface)–$p_{6}$ (output port). These three connection pairs cannot share channels, and the corresponding channels between them must not cross; we define these as mutually exclusive connection pairs.

**Figure 3:**
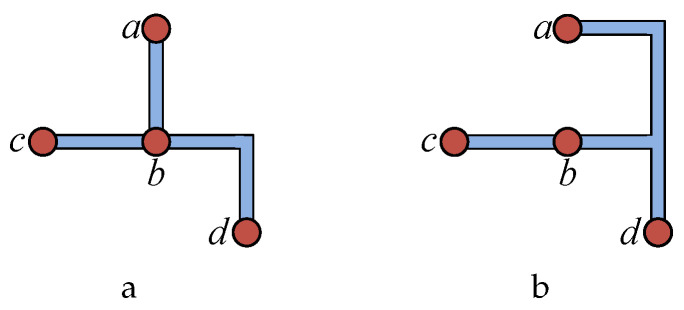
(**a**) The routing result that minimizes the path length. (**b**) The routing result that balances the path length and transportation task conflicts.

Routing optimization for CFMBs must not only minimize the total channel length and the number of intersections but also address time conflicts between fluid transportation tasks. The example in Table 1 highlights this trade-off: (1,3) indicates that the fluid transportation task requires the use of the flow channel between node *a* and node *b* within a 1–3 s interval. If the objective is solely to minimize total channel length (as shown in Figure 3a), conflicts will arise between the fluid transportation tasks of connection pair $Con_{2}$ (path b→c) and $Con_{3}$ (path a→b→d). By sacrificing some channel length, as illustrated in Figure 3b, these conflicts can be avoided, thus reducing the total conflict time between tasks. Therefore, the routing phase must balance the manufacturing costs of CFMBs (total channel length and number of intersections) with the total conflict time between fluid transportation tasks. In CFMBs, components are used to perform specific operations (such as mixers for mixing different input fluids). In the routing problem, components are viewed as obstacles; channels cannot pass through the interior of components but can be arranged along component edges. Each connection pair consists of two nodes and is bound to one or more fluid transportation tasks (as shown in Table 1).

### 3.2. Basic Concept and Design Challenges of Application Mapping

Given the binding and scheduling results of high-level synthesis, physical design outcomes, and the biochemical reaction sequence graph G(O,E), we aim to generate an optimized application mapping scheme ϕ=〈T,F〉. Here, *O* represents the set of operations, and *E* represents the dependencies between operations, where $e_{i,j}\in E$ indicates that operation $o_{i}$ is the predecessor of operation $o_{j}$, meaning that the output of $o_{i}$ serves as the input to $o_{j}$. T denotes the scheduling results of the application mapping, and F represents the binding results. Specifically, $T=\{T_{o},T_{e},T_{R}\}$, where $T_{o}$ represents the scheduling time set of operations, $T_{e}$ represents the scheduling time set of fluid transportation tasks, and $T_{R}$ represents the scheduling time set of wash operations. Similarly, $F=\{F_{o},F_{e},F_{R}\}$, where $F_{o}$ represents the binding relationship between operations and functional components, $F_{e}$ represents the binding relationship between fluid transportation tasks and specific flow paths, and $F_{R}$ represents the binding relationship between wash operations and flow paths.

It is important to note that there is a fundamental difference between application mapping and the binding and scheduling results of high-level synthesis: (1) the scheduling results at the high-level synthesis stage are only estimates of time, while application mapping determines the specific execution time, and (2) the high-level synthesis stage binds the flow path types, while the application mapping stage binds the specific physical channels. The primary goal of application mapping is to minimize the total completion time of biochemical reactions. For each operation $o_{i}$, we define three critical time points: ready time $t_{ready}\left(o_{i}\right)$, start time $t_{start}\left(o_{i}\right)$, and finish time $t_{finish}\left(o_{i}\right)$. The ready time indicates the earliest time the operation can start. The ready time of operation $o_{i}$ depends on the finish time of all its predecessor operations, while the start and finish times are determined during scheduling. These three time points must satisfy the following:

(1) $$t_{ready}\left(o_{i}\right)\leq t_{start}\left(o_{i}\right)<t_{finish}\left(o_{i}\right)$$

When operation $o_{i}$ is a predecessor of operation $o_{j}$ and they are bound to different functional components, the system must execute a fluid transportation task to transfer fluid between the bound components. If both operations are bound to the same component, no additional transportation task is required. It is important to emphasize that wash operations are not explicitly represented in the sequence graph but are dynamically determined during execution based on the contamination status of the required flow paths for the current task. For each wash operation $w_{i}$, its start time and finish time are $t_{start}\left(w_{i}\right)$ and $t_{finish}\left(w_{i}\right)$, respectively. Thus, the execution time of the wash operation is calculated as

(2) $$t_{exe}\left(w_{i}\right)=t_{prep}+t_{dis}\left(w_{i}\right)+\frac{len(w_{i})}{v_{p}}$$

where $t_{prep}$ represents the preparation time, which is the time required for the valves in the chip to switch to the specified state and make the corresponding flow path ready to guide the buffer fluid to the contaminated channels or components. It is worth noting that the valve state switching can usually be completed in milliseconds [29,30]. $t_{dis}$ represents the pollutant dissolution time. Since pollutants attached to the walls/components of the channel cannot be completely removed by liquid convection alone, the buffer fluid needs to remain at the contamination spots for a sufficient duration to dissolve the pollutants, which are then removed with the buffer fluid. The dissolution time of pollutants is mainly determined by their diffusion coefficient—the smaller the diffusion coefficient, the longer the dissolution time. For example, small molecules such as a lysis buffer have a high diffusion coefficient (around $10^{-5}\;{\mathrm{cm}}^{2}/s$) and can typically dissolve within a short time (about 0.2 s) [31], while larger molecules, such as tobacco mosaic virus, have a lower diffusion coefficient (around $5\times 10^{-8}\;{\mathrm{cm}}^{2}/s$), and take a relatively longer time (about 6 s) to dissolve sufficiently [31]. $len(w_{i})$ represents the length of the wash path, and $v_{p}$ represents the fluid flow rate.

In the current model, the execution time of wash operations is estimated using a generalized model that considers three components: valve preparation time, dissolution time, and flushing time. While we incorporate the dissolution time based on diffusion coefficients of contaminants (e.g., small molecules like a lysis buffer vs. larger molecules like viruses), the model does not yet differentiate between Newtonian and non-Newtonian fluids or between low- and high-viscosity fluids. In practical microfluidic systems, fluids such as oils or emulsions can exhibit significantly prolonged washing periods, even when using clearing agents like isopropyl alcohol. The current model simplifies these variations to preserve computational tractability. As part of future work, we plan to incorporate fluid-aware washing models where fluid properties—such as viscosity, interfacial tension, and rheological behavior—affect the washing cost dynamically. This can be achieved by extending our framework to support fluid-specific delay penalties or lookup-based timing models calibrated from experimental data.

The application mapping stage must satisfy the following key constraints:

Component mutual exclusion: A component cannot execute two operations simultaneously. If two operations $o_{i}$ and $o_{j}$ are bound to the same component, i.e., $F_{o}\left(o_{i}\right)=F_{o}\left(o_{j}\right)$, then they must satisfy

(3) $$t_{finish}\left(o_{i}\right)\leq t_{start}\left(o_{j}\right)\;\mathrm{or}\;t_{finish}\left(o_{j}\right)\leq t_{start}\left(o_{i}\right)$$

Operation dependency: For each dependency $e_{i,j}\in E$, operation $o_{i}$ must be completed first, followed by a transportation task, and then operation $o_{j}$ is executed on the target component. That is, it must satisfy

(4) $$t_{finish}\left(o_{i}\right)\leq t_{start}\left(e_{i,j}\right)\leq t_{finish}\left(e_{i,j}\right)\leq t_{start}\left(o_{j}\right)$$

Channel mutual exclusion: Two different flow paths bound to the same channel or component cannot use that channel or component simultaneously. If $F_{e}\left(e_{i,j}\right)\cap F_{e}\left(e_{k,l}\right)\neq \emptyset$, then it must satisfy

(5) $$t_{finish}\left(e_{i,j}\right)\leq t_{start}\left(e_{k,l}\right)\;\mathrm{or}\;t_{finish}\left(e_{k,l}\right)\leq t_{start}\left(e_{i,j}\right)$$

Contamination handling: When a component *m* is contaminated, it cannot be used to execute any operation. If component *m* is required to execute operation $o_{j}$, a wash operation $w_{i}$ must be executed first, and the flow path used for the wash operation must include component *m*, ensuring

(6) $$t_{start}\left(w_{i}\right)<t_{finish}\left(w_{i}\right)\leq t_{start}\left(o_{j}\right)$$

Similarly, when a channel is contaminated, if it is necessary to use that channel for fluid transportation, a wash operation must be performed to remove the contaminants from the channel before performing the transportation task.

Existing work typically only performs single-round scheduling, which may result in unreasonable operation scheduling sequences, leading to conflicts between fluid transportation tasks and an increase in wash times, ultimately prolonging the total completion time of biochemical reactions. The following case will elaborate on this issue in detail.

**Figure 4:**
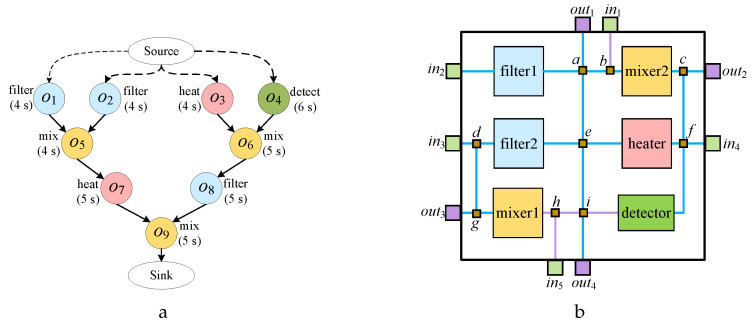
(**a**) Sequencing graph of a bioassay. (**b**) The flow-layer chip layout designed for the bioassay in (**a**).

**Figure 5:**
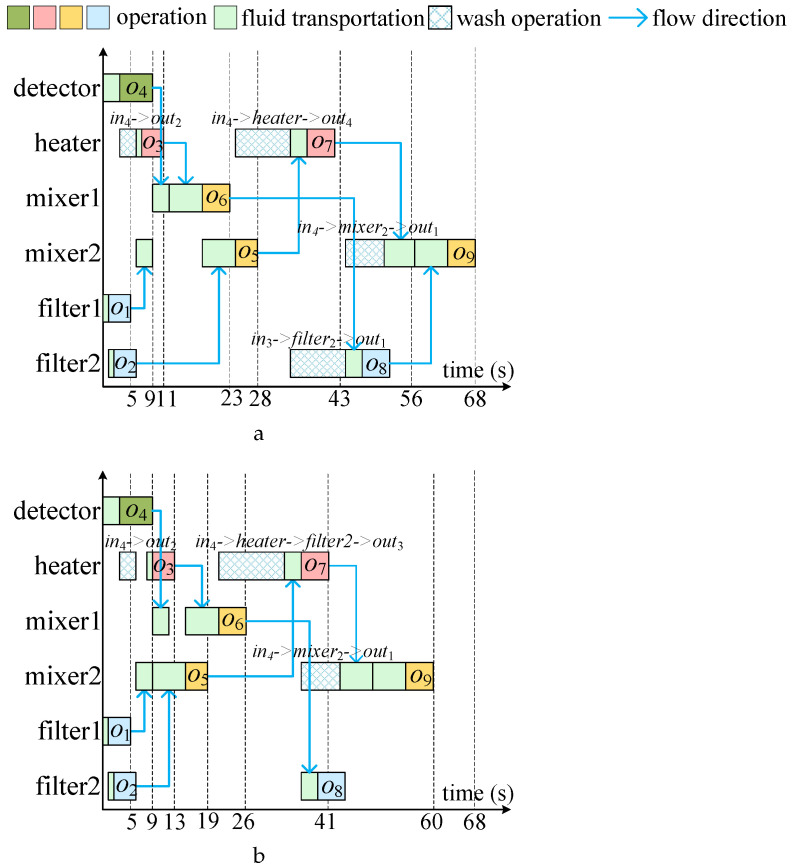
Two scheduling schemes for mapping the bioassay described in Figure 4a onto the chip layout shown in Figure 4b. (**a**) A scheduling with a relatively long completion time (68 s). (**b**) An optimized scheduling with a shorter completion time (60 s).

Figure 4a shows a typical biochemical reaction sequence graph, Figure 4b presents the flow-layer chip layout designed for the bioassay in (a). Figure 5a presents the application mapping scheduling scheme proposed in reference [16], which uses a list scheduling algorithm to sort operations. Specifically, for the current operation $o_{i}$ to be scheduled, the algorithm binds the earliest available component *m* and uses the Dijkstra algorithm to compute the optimal flow path required for fluid input. If the flow path or component *m* is contaminated, a wash operation is triggered to eliminate the contamination, followed by a transportation task to deliver fluid to component *m*. The algorithm schedules operations on the order of $o_{4},o_{3},o_{1},o_{2},o_{6},o_{5},o_{7},o_{8},o_{9}$, and the specific components bound are shown in Figure 5a. However, this scheduling method adopts a greedy-based scheduling order, causing fluid transportation conflicts between the input tasks of operations $o_{5}$ and $o_{6}$, which delays the execution of $o_{5}$. Additionally, this method employs a demand-driven wash optimization strategy, which means wash operations are only triggered when component or channel binding fails, ultimately resulting in four wash tasks that delay the total execution time of the biochemical reaction by 68 s.

The main flaw of existing work lies in neglecting the valuable information generated during the first round of scheduling and failing to perform multi-round optimized scheduling, resulting in no further improvement in the quality of the application mapping scheme. In fact, after the completion of the first round of scheduling, we can obtain detailed scheduling information about the interactions between operations. By analyzing this information, we found that there are conflicts between the fluid input tasks of operations $o_{5}$ and $o_{6}$, and that the execution times of the wash operations for the heater and filter2 are close. If a wash path can be found that passes through both components, synchronized washing can be achieved, significantly reducing the total wash time.

Figure 5b shows the optimized results obtained by adjusting the operation order using scheduling information. From the figure, it is clear that by adjusting the operation order to $o_{4}$, $o_{1}$, $o_{2}$, $o_{5}$, $o_{3}$, $o_{6}$, $o_{7}$, $o_{8}$, $o_{9}$, the total conflict time between fluid transportation tasks is reduced. Additionally, by using the same wash path to simultaneously clean both the heater and filter2, the number of wash tasks is reduced to three, thereby effectively reducing the total wash time. Due to the more reasonable operation sequence and the optimization of wash task merging, the multi-round scheduling strategy effectively reduced the total completion time of the biochemical reaction to 60 s.

### 3.3. Problem Formulation

Based on the analysis above, the path-driven routing and scheduling problem for CFMBs can be formulated as follows:

*Inputs*: (1) a bioassay modeled as a sequencing graph; (2) binding and scheduling results of high-level synthesis; and (3) placement results of components and ports.

*Outputs*: the scheduling results T and the binding results F of the application mapping.

*Objectives*: (1) Reducing the completion time of the bioassay; (2) reducing the total length of flow channels in the generated chip.

## 4. Details of Proposed Design Flow

In this section, we propose a systematic design flow to effectively address the problem formulated in Section 3.3. A path-driven integrated design framework is proposed for CFMBs that organically combines two key stages: routing and application mapping, with flow-path optimization as the core objective. The main innovations of this framework include (1) the simultaneous optimization of total channel length, number of intersections, and conflict frequency in fluid transportation tasks; (2) the proactive consideration of application mapping requirements during the routing stage; and (3) the implementation of multiple iterative optimizations in the application mapping stage using routing information.

### 4.1. Path-Driven Conflict-Aware Routing

This section presents a path-driven conflict-aware routing algorithm for CFMBs, designed to optimize three key metrics: total channel length, number of intersections, and conflict frequency in fluid transportation tasks. The algorithm implementation consists of four sequential steps. First, conflict-aware routing initialization is conducted to reduce the number of connection pairs and generate a random initial population. Second, routing preprocessing is performed to pre-calculate essential information about connection pairs and task overlap times, eliminating redundant computations. The third step employs a hybrid particle swarm optimization-based conflict-aware routing algorithm (HPSO-CAR), which enhances search capabilities by incorporating orthogonal experimental design (OED) into genetic algorithm crossover operations and integrating these within the particle swarm framework. In the fourth step, results undergo adjustment to ensure channels properly bypass components and prevent shared or crossed channels between mutually exclusive connection pairs. Through this four-step collaborative approach, the algorithm effectively balances physical constraints and performance requirements, addressing the complex challenges inherent in CFMB routing design.

#### 4.1.1. Initialization and Preprocessing Phase

During the initialization phase, the system must complete two key tasks: (1) reducing loops formed by connection pairs to enhance channel reuse (2) and initializing the particle swarm. Connection pairs may form loop structures; for example, three connection pairs, (a,b), (b,c), and (c,a), create a cycle. If the execution time intervals of the corresponding fluid transportation tasks do not significantly overlap, one of the connection pairs can be removed, allowing its task to be executed through the channels formed by the remaining two connection pairs. For instance, removing the connection pair (c,a) enables the reuse of the flow path formed by (a,b) and (b,c) to transport fluid between nodes *c* and *a*. For loops formed by multiple connection pairs, we identify the connection pair Cn with the minimum total conflict time with other pairs. If the total conflict time $T_{1}$ between Cn and all other connection pairs in the loop is less than a threshold *T*, then Cn is removed, and its fluid transportation task is reassigned to other connection pairs. Otherwise, all connection pairs are retained. The choice of threshold *T* is critical: a value that is too large increases the conflict time of fluid transportation tasks, while a value that is too small prevents channel reuse, increasing the cost of CFMBs. Since precise transportation time depends on the routing result and flow rate, we assume that each fluid transportation task lasts 3 s during the preprocessing stage for conflict estimation. This estimation is based on a default flow rate of 10 mm/s, which is consistent with the flow rate used in the application mapping phase.

To optimize the system, all nodes and components are assigned unique identifiers. Suppose the total number of connection pairs is *n*; then the particle dimension is also *n*. The encoding format of a particle is $p_{1}\;q_{1}\;c_{1}\;p_{2}\;q_{2}\;c_{2}\;\ldots \;p_{n}\;q_{n}\;c_{n}$, where $p_{i}\;q_{i}\;c_{i}$ represents the connection mode of the *i*-th connection pair, $p_{i}$ is the source node, $q_{i}$ is the destination node, and $c_{i}$ represents the selected connection type between these nodes. During initialization, $p_{i}$ and $q_{i}$ are set as the source and destination nodes of the *i*-th connection pair, while $c_{i}$ is randomly chosen from four possible connection options [32]. After testing, the particle swarm size is set to 100. Once the particle swarm is initialized, each particle updates its velocity and position iteratively. This process requires frequent computations of three types of information: (1) the obstacle-crossing condition of each routing edge; (2) crossovers between different connection pairs under different selection modes; and (3) the estimated total conflict time when connection pairs intersect. To improve algorithm efficiency, we compute and store these data using hash tables during preprocessing:

- Obstacle traversal table: Let $p=\{p_{1},p_{2},\ldots ,p_{n}\}$ be the set of nodes and $b=\{b_{1},b_{2},\ldots ,b_{m}\}$ be the set of components. For the *k*-th connection pair $\left(p_{i},p_{j}\right)$, the number of components it traverses under different connection modes is computed, and all traversed components are recorded as the set $B_{k}^{*}=\left\{b_{k1},b_{k2},\ldots ,b_{kn}\right\}$.
- Crossover calculation: The intersections of all connection pairs under different selection modes are computed.
- Conflict time calculation: The conflict duration between fluid transportation tasks of different connection pairs is recorded. For instance, if connection pair $Cn_{1}$ operates during time intervals (0,2) and (4,6), and $Cn_{2}$ operates during (4,6) and (16,18), the conflict duration between them is 2 s.

These preprocessing and optimization measures enable the system to perform subsequent particle swarm optimization calculations more efficiently, significantly reducing the algorithm’s runtime.

#### 4.1.2. Hybrid Particle Swarm Optimization

In this step, the algorithm integrates the OED method into the genetic algorithm’s crossover operator and incorporates crossover and mutation operators into the particle swarm algorithm, achieving efficient particle updates. Although we adopt a swarm-based routing strategy inspired by particle behaviors, our model does not treat fluid as discrete particles in a physical sense. Instead, all fluid transport is modeled under the continuum assumption, which is widely applicable at the micrometer scale in continuous-flow microfluidic systems, as discussed in [33]. The “particle swarm” term refers solely to the scheduling heuristic, not to a discretized fluid model.

We choose the HPSO algorithm as the core method for solving the conflict-aware routing problem in microfluidic chips, mainly because of its excellent multi-objective optimization capability, which enables effective trade-offs among channel length, the number of intersections, and conflict time. At the same time, HPSO is suitable for handling the large-scale discrete combinatorial space involved in routing and features parallel search capabilities, which can improve solving efficiency and is particularly suitable for the initial routing of complex connection pairs. Compared with traditional algorithms, HPSO also supports the flexible adjustment of weight parameters to accommodate different optimization priorities in microfluidic applications. Additionally, by introducing a penalty function mechanism, it effectively balances optimization objectives and design constraints, ensuring the feasibility and quality of the solution.

This study innovatively integrates OED and genetic operators into the HPSO algorithm framework, significantly enhancing the performance of microfluidic chip routing optimization from multiple dimensions. On one hand, OED guides particles to perform orthogonal learning from multiple high-quality solutions, improving optimization efficiency and reducing routing conflicts caused by inter-dimensional dependencies. On the other hand, genetic operators enhance the exploration ability of the solution space, effectively maintaining solution diversity and preventing premature convergence. In addition, the algorithm achieves a dynamic balance between “exploration and exploitation” through adaptive adjustment of inertia weight and crossover probability. Overall, the HPSO-OED-GA hybrid framework not only improves solution quality and efficiency but also enhances the adaptability to complex trade-offs between routing constraints and multiple objectives, providing strong support for generating high-quality, low-conflict microfluidic channel layouts. To facilitate the understanding of the mathematical expressions and symbols used in this section, we provide a list of notations in Nomenclature.

The mutation operation is as follows: assuming the particle encoding is $p_{1}\;q_{1}\;c_{1}\;p_{2}\;q_{2}\;c_{2}\;\ldots \;p_{n}\;q_{n}\;c_{n}$, we first randomly generate a random number *i* in the interval [1,n], then execute the operation *a* with probability $h_{1}$, and with probability $1-h_{1}$ execute operation *b*:

(a) Randomly change the $c_{i}$ value in the particle encoding, updating the connection selection method of the *i*-th connection pair.

(b) Select the *i*-th connection pair, where the source node is $p_{i}$ and the destination node is $q_{i}$. If there exists a node *x* that is connected to node $q_{i}$, then set $p_{i}$ in $p_{i}\;q_{i}\;c_{i}$ of the particle encoding to *x*, changing the path of the *i*-th connection pair to $p_{i}$-*x*-$q_{i}$, maintaining connectivity between $p_{i}$ and $q_{i}$.

The literature [34] integrates the OED method into the PSO algorithm, forming an orthogonal learning (OL) strategy. This strategy constructs guide particles $P_{o}$ based on the particle’s historical best position $P_{b}$ and the neighborhood’s historical best position $G_{b}$ to guide particle updates. The particle update method is shown as follows:

(7) $$v_{i}^{t}=\omega \cdot v_{i}^{t-1}+c\cdot r\cdot \left(P_{o}^{t-1}-x_{i}^{t}\right)$$

where ω is the inertia weight, *c* is the acceleration factor, and *r* is a random number in the range [0,1].

Since the routing problem in this section belongs to discrete PSO problems, the above particle update method cannot be used directly. This section integrates the OED method into the particle’s crossover process. Let the current particle be $P_{a}$, with each dimension of the particle corresponding one-to-one with connection pairs, where the *i*-th dimension corresponds to the connection method of the *i*-th connection pair.

For the *i*-th dimension of $P_{a}$, if it is consistent with $P_{o}$, then the *i*-th dimension remains unchanged; otherwise, under the premise of not affecting the connectivity between the source node and target node of the *i*-th connection pair, the *i*-th dimension of $P_{a}$ is set to the *i*-th dimension of $P_{o}$ with a certain probability. For example, if particle $P_{a}$ is 123-230-352-241 and particle $P_{o}$ is 142-230-351-342, both have four dimensions. Among them, the first, third, and fourth dimensions of $P_{a}$ and $P_{o}$ are different. If the third dimension remains unchanged, the updated particle would be 142-230-352-342.

Each particle corresponds to a routing solution, and its fitness value is related to the total channel length, number of intersections, and total conflict time $T_{\mathrm{total}}$ of the routing. The calculation of $T_{\mathrm{total}}$ is shown as follows:

(8) $$T_{\mathrm{total}}=\sum_{i=1}^{n}\sum_{\begin{array}{c} j=1\\ j\neq i \end{array}}^{n}\left(OLT\left(C_{i},C_{j}\right)\cdot IsC\left(C_{i},C_{j}\right)\right)$$

where $OLT(C_{i},C_{j})$ is the overlap time between the transportation tasks of connection pair $C_{i}$ and connection pair $C_{j}$. IsC is a 0–1 variable; if the channels formed by connection pair $C_{i}$ and connection pair $C_{j}$ have an overlap or intersection, its value is 1; otherwise, it is 0.

The fitness function is shown as follows:

(9) $$\mathrm{Fitness}\left(X_{i}\right)=\alpha \cdot L+\beta \cdot CN+\gamma \cdot T_{\mathrm{total}}$$

where CN is the number of intersections, *L* is the total channel length, and $T_{\mathrm{total}}$ is the total conflict duration between different connection pairs. The coefficients α,β,γ are determined through experiments as α=1, β=15, γ=15.

The OED method uses pre-built orthogonal arrays (OA) to design experimental combinations to discover optimal combinations. Let the global best particle be $G_{b}$ and the historical best particle $P_{a}$ be $P_{b}$. The guide particle $P_{o}$ for particle $P_{a}$ is constructed using the OED method from $G_{b}$ and $P_{b}$. All particles have the same dimension, which is the number of connection pairs *D*. For a two-level *D*-dimensional orthogonal experimental design, OA satisfies the following properties: (a) OA is a binary matrix, with element values of 0 or 1, and (b) the number of occurrences of 0 and 1 in each column is equal.

The OA construction method is as follows: The number of rows $M=2^{\left\lceil \log_{2}\left(D+1\right)\right\rceil }$, and the number of columns is *D*. Let $u=\log_{2}\left(M\right)$. First, construct the elements with column numbers that are powers of 2, as shown in Equation (10):

(10) $$L\left[a\right]\left[b\right]=\left(\frac{a-1}{2^{u-k}}\right)\;\mathrm{mod}\;2$$

where a=1,2,…,M, $b=2^{k-1}$, k=1,2,…,u. Other elements are constructed as shown in Equation (11):

(11) L[a][b+s]=(L[a][s]+L[a][b])mod2

where a=1,2,…,M, $b=2^{k-1}$, s=1,2,…,b−1, k=1,2,…,u.

The construction method for guide particle $P_{o}$ is as follows:

1. Construct M×D orthogonal matrix OA.
2. Construct experimental particles $X_{i}$. For the *j*-th dimension of $X_{i}$, if the value of the *i*-th row and *j*-th column of OA is 1, then select the *j*-th dimension of $P_{b}$ as the value; otherwise, select the *j*-th dimension of $G_{b}$ as the value. i∈[1,M],j∈[1,D].
3. Based on *M* experimental particles, conduct orthogonal experiments to construct particle $P_{x}$. Calculate the fitness value of each $X_{i}$, and compute the $S_{jq}$ value (the score of the *q*-th level of the *j*-th dimension):
  (12) $$S_{jq}=\frac{\sum_{i=1}^{M}\mathrm{Fitness}\left(X_{i}\right)\cdot k_{ijq}}{\sum_{i=1}^{M}k_{ijq}}$$
  where $\mathrm{Fitness}(X_{i})$ is the fitness value of $X_{i}$, and $k_{ijq}$ is a 0–1 variable, which is 1 when the value of the *i*-th row and *j*-th column of OA is *q*; otherwise, it is 0.
4. If $S_{j0}>S_{j1}$, select the *j*-th dimension of $P_{b}$ as the value of the *j*-th dimension of $P_{x}$; otherwise, select the *j*-th dimension of $G_{b}$ as the value.
5. Compare the fitness of the particle $X_{ib}$ with the best fitness in the set $\left\{X_{i}\right\}$ and the fitness of $P_{x}$, and select the better one as the guide particle $P_{o}$.

Other works often have particles learn first from the individual cognitive component and then from the social cognitive component after mutation. However, this may lead to oscillation phenomena, reducing the algorithm’s search capability [34]. This section applies the OED method to discrete PSO problems, constructing guide particles based on the individual historical best and the global historical best, and performs crossover between mutated particles and guide particles. The particle update method is shown as follows:

(13) $$X_{i}^{t}=F_{3}\left(F_{1}\left(X_{i}^{t-1},\omega \right),F_{2}\left(P_{b},G_{b}\right),h_{2}\right)$$

where ω represents the inertia weight, $F_{1}$ represents the mutation operation (also representing the particle’s velocity), $F_{2}$ represents the OED operation, and $F_{3}$ represents the crossover operation (also representing the particle’s cognitive component). $P_{b}$ is the individual historical best, and $G_{b}$ is the global historical best. $h_{2}$ is a probability value. The parameters ω and $h_{2}$ change linearly during the iteration process, calculated as shown in Equations (14) and (15), respectively:

(14) $$\omega =\omega_{\mathrm{start}}-\frac{\omega_{\mathrm{start}}-\omega_{\mathrm{end}}}{\mathrm{iterators}}\cdot it$$

where $\omega_{\mathrm{start}}$ is the initial value of ω, $\omega_{\mathrm{end}}$ is the final value, iterators is the total number of iterations, and it is the current iteration round.

(15) $$h_{2}=h_{\mathrm{start}}-\frac{h_{\mathrm{start}}-h_{\mathrm{end}}}{\mathrm{iterators}}\cdot it$$

where $h_{\mathrm{start}}$ is the initial value of $h_{2}$, and $h_{\mathrm{end}}$ is the final value.

The particle’s velocity update is shown in Equation (16):

(16) $$V_{i}^{t}=F_{1}\left(X_{i}^{t-1},\omega \right)=\left\{\begin{array}{cc} M(X_{i}^{t-1}), & \mathrm{if}\;r_{1}<\omega \\ X_{i}^{t-1}, & \mathrm{otherwise} \end{array}\right.$$

The guide particle $P_{o}$ constructed by the OED operation is represented as

(17) $$P_{o}=F_{2}\left(X_{1},X_{2}\right)=\left\{\begin{array}{cc} X_{1}, & \mathrm{if}\;S_{j0}<S_{j1}\\ X_{2}, & \mathrm{otherwise} \end{array}\right.$$

where $X_{1}$ and $X_{2}$ are two different particles, and the calculations of $S_{j0}$ and $S_{j1}$ are shown in Equation (12).

Particles learn from the particle $P_{o}$ constructed by the individual best and the global best, and its cognitive component is represented as

(18) $$X_{i}^{t}=F_{3}\left(C_{i}^{t},P_{o},h_{2}\right)=\left\{\begin{array}{cc} P_{o}, & \mathrm{if}\;r_{2}>h_{2}\\ C_{i}^{t}, & \mathrm{otherwise} \end{array}\right.$$

where $h_{2}$ represents the crossover probability between the current particle and the guide particle.

#### 4.1.3. Particle Adjustment in HPSO-CAR

After particles complete their flight, the routing solution corresponding to the global best particle obtained by HPSO-CAR may pass through components that do not meet the constraint conditions. Therefore, this step needs to use obstacle avoidance strategies to adjust channels that pass through components, making them bypass all components. Additionally, it is necessary to prevent channels corresponding to mutually exclusive connection pairs from sharing or crossing each other. Thus, after obstacle avoidance, path planning strategies are needed to avoid this situation.

First, channels that traverse components (i.e., obstacles) need to be adjusted to bypass components. If changing the selection can bypass components, then change the selection; if changing the selection alone cannot bypass components, then select one or multiple corner points of components as transit points to bypass components. The specific process of the obstacle avoidance strategy is as follows [32]:

1. For the edge $p_{i}\;q_{i}\;c_{i}$ corresponding to the *i*-th dimension of the particle, determine whether it intersects with any obstacle components by consulting the obstacle traversal table. If the edge avoids all obstacles, proceed to examine the next edge; otherwise, execute step (2).
2. If all components can be avoided through selection 0 or selection 1, use the corresponding selection to replace the original selection $c_{i}$; otherwise, execute step (3).
3. Determine all components that $p_{i}\;q_{i}\;c_{i}$ passes through via the obstacle traversal table, and sort them in non-descending order according to the distance from $p_{i}$ to the center points of these components. Let the arrangement table of traversed components be $C_{k}=\left\{C_{k,1},C_{k,2},\ldots ,C_{k,n}\right\}$. Let the starting point $s=p_{i}$ and the component that currently needs to be avoided be $C=C_{k,j}$, j=1; then, execute step (4).
4. Select the corner point cp from component *C* that is closest to the perpendicular distance from the straight line $sq_{i}$, and calculate the connection method between point *s* and corner point cp. Priority is given to selection 0 or selection 1; if both these selections pass through components, use selection 2 or selection 3, and add its component traversal information to the obstacle traversal table. Let s=cp, j=j+1. If j=n, execute step (5); otherwise, continue to execute step (4).
5. Calculate the connection information between *s* and $q_{i}$, and add its component traversal information to the obstacle traversal table.

**Figure 6:**
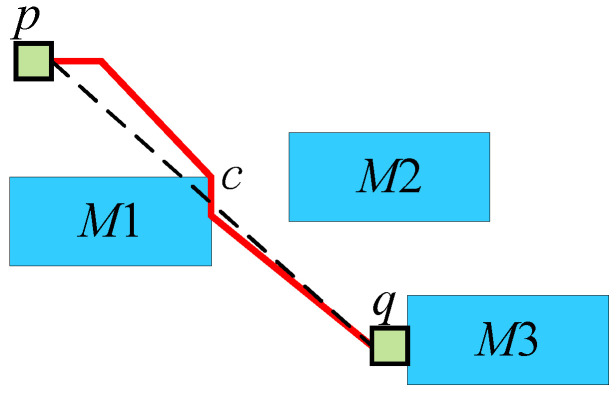
Addition of extra points for obstacle avoidance.

Figure 6 shows an example of the fourth step above. Points *p* and *q* cannot avoid passing through components using all four connection methods. At this point, set *p* as *s*, and the point closest to the straight line sq (shown as a dotted line in the figure) is the corner point *c* at the upper right of component M1. At this time, use selection 0 to connect sc (shown by the red line in the figure). Subsequently, set *c* as *s*, use selection 0 to connect sq, and complete the obstacle avoidance.

Additionally, mutually exclusive connection pairs cannot have crossings or channel sharing. To solve this problem, a path planning strategy is proposed with the following specific steps:

1. Construct a graph G(V,E), where $v_{i}\in V$ represents the intersections and nodes that exist in the CFMBs after executing the obstacle avoidance step and $e_{i}\in E$ represents the connection relationship between nodes $v_{i}$ in the graph, and record the set of edges used by each connection pair.
2. Calculate the crossing situations between all mutually exclusive connection pairs, and record the connection pairs that produce mutual exclusion in the mutual exclusion table.
3. For mutually exclusive connection pairs $\left(Cn_{1},Cn_{2}\right)$ that produce crossings or channel sharing, use the DFS algorithm to calculate their minimum-conflict paths ${\mathrm{path}}_{1}$ and ${\mathrm{path}}_{2}$ from the source node to the destination node, respectively. The minimum-conflict path is the path with the minimum conflict time among the set of paths from the source node to the destination node. If the conflict time of ${\mathrm{path}}_{1}$ with other connection pairs is less than or equal to the conflict time of ${\mathrm{path}}_{2}$ with other connection pairs, modify the flow path of $Cn_{1}$ to ${\mathrm{path}}_{1}$; otherwise, modify the flow path of $Cn_{2}$ to ${\mathrm{path}}_{2}$.

When a connection pair Cn uses a flow path, it will produce sharing or crossing with the paths used by other connection pairs. The total conflict time of Cn is the sum of the conflict times between Cn and these connection pairs. When searching for the flow path of Cn using the DFS algorithm, the flow path that minimizes the total conflict time of Cn in the solution space is the minimum-conflict path.

### 4.2. Path-Driven Application Mapping Algorithm

During the high-level synthesis stage, each operation has been assigned high-quality binding results, and corresponding fluid transportation tasks have been determined based on these bindings. Subsequently, in the physical design stage, placement and routing are performed according to these specific transportation tasks, with proactive consideration of potential conflicts among fluid transportation paths arising during the application mapping stage, thus achieving a high-quality flow-layer chip architecture. Therefore, unlike the greedy method employed in [16], the application mapping algorithm in this section does not greedily select components during execution. Instead, it selects the components for executing operations based on the binding results determined earlier and utilizes flow paths derived from the physical design stage to facilitate fluid transportation tasks.

The detailed path-driven iterative application mapping algorithm is presented in Algorithm 1. In this algorithm, $T_{min}$ denotes the minimum biochemical reaction completion time across multiple iterations. If $T_{min}$ does not improve for three consecutive iterations, the flag is set to false (lines 19–20), and the iteration terminates. At the start of each iteration, operations without parent dependencies are initially added to the ready queue *Q* (line 3). When an operation is completed, the in-degree of its child operations is decremented by one; once a child operation’s in-degree becomes zero, it is placed into queue *Q*. Meanwhile, queue $Q^{\prime }$ contains operations scheduled for immediate execution.

During scheduling, if the queue $Q^{\prime }$ is not empty, the operation at the front of $Q^{\prime }$ is dequeued and scheduled. Otherwise, operations from queue *Q* are transferred to queue $Q^{\prime }$ after being sorted using an operation-sorting algorithm. For example, in the sequencing graph depicted in Figure 4a, at the start of each iteration, queue $Q^{\prime }$ is initially empty, and queue *Q* contains operations $o_{1}$, $o_{2}$, $o_{3}$, and $o_{4}$, which have no parent operations. At this point, the sorting algorithm is invoked to sort elements in *Q*, and the sorted operations are then added to $Q^{\prime }$. After operations $o_{1}$ and $o_{2}$ complete their execution, operation $o_{5}$ is added to *Q*. Similarly, after operations $o_{3}$ and $o_{4}$ complete their execution, operation $o_{6}$ is added to *Q*. Once operations $o_{1}$ through $o_{4}$ are executed and $Q^{\prime }$ becomes empty, the sorting algorithm is again called to transfer sorted operations from *Q* into $Q^{\prime }$.

The operation sorting algorithm aims to reduce conflicts between fluid transportation tasks in CFMBs. The algorithm receives the set of currently ready operations OS and the scheduling result of the previous round as input and outputs the operation execution order that minimizes conflicts. The algorithm first constructs an undirected graph $G^{\prime }\left(O,CE\right)$ to represent the conflict relationship between operations, where *O* represents the operation set and CE represents the edges between non-conflicting operation pairs. Two operations $o_{i}$ and $o_{j}$ are considered to have a conflict when one of the following conditions is met: (1) in the scheduling results generated in the high-level synthesis phase, the fluid transportation tasks of the two operations overlap in time and share channels or ports in the flow path; (2) in the previous round of scheduling, a fluid transportation task of one operation is delayed due to sharing a channel with another operation. Specifically, if the transportation task of operation $o_{i}$ is $task_{1}$ and the transportation task of operation $o_{j}$ is $task_{2}$, when the intersection of $\left[t_{ready}\left(task_{1}\right),t_{ready}\left(task_{1}\right)+t_{c2}\right]$ and $\left[t_{ready}\left(task_{2}\right),t_{ready}\left(task_{2}\right)+t_{c2}\right]$ is non-empty, $o_{i}$ and $o_{j}$ are considered to have a conflict; otherwise, an edge $ce_{i,j}$ is added to the graph. After graph construction, the algorithm proceeds with sorting through two nested loops: the outer loop selects the operation with the highest priority from OS and adds it to stack sta; the inner loop takes operations from sta and adds them to the result queue $Q^{\prime }$, while adding neighboring nodes of the operation to the stack in ascending order of priority. The operation priority is calculated by the formula $q\left(o_{j}\right)=t_{e}\left(o_{j}\right)+H_{j}\cdot \left(\max\left(q_{i}\right)+t_{tr}\left(m_{1},m_{2}\right)\right)$, where $t_{e}\left(o_{j}\right)$ is the execution time of operation $o_{j}$, $H_{j}$ is a binary variable (0 indicates no child operations, 1 indicates having child operations), $\max(q_{i})$ is the maximum priority among $o_{j}$’s child operations, and $t_{tr}\left(m_{1},m_{2}\right)$ is the estimated transport time of liquid from component $m_{1}$ bound to $o_{j}$ to component $m_{2}$ bound to $o_{i}$. Through this approach, the algorithm effectively reduces conflicts between transportation tasks and improves the scheduling efficiency of CFMBs.

Before executing a specific operation $o_{i}$, the input fluids required by this operation must be transported to their bound destination component *m*. It is necessary to ensure that the component *m* is ready, meaning it contains no unnecessary fluid and is free from contamination. If component *m* does contain unnecessary fluid, it must be transported elsewhere on the biochip (lines 9–10). Suppose the fluid in the component is the output of a completed operation $o_{i}$, and let the child operation of $o_{i}$ be $o_{k}$, where $o_{k}\neq o_{j}$. If the current component $m^{\prime }$ bound to $o_{k}$ is uncontaminated and free of fluid (if contamination is present, a wash operation is initiated), the fluid in *m* is transported to $m^{\prime }$. When $o_{k}=o_{i}$, the fluid in *m* is already required for the current operation, so no transportation is necessary. Otherwise, the fluid from *m* needs to be transported to a dedicated storage unit.

When transporting the required fluids for operation $o_{i}$ to component *m*, it is crucial to ensure that the flow path $p_{f}$ remains uncontaminated. Initially, the segments requiring cleaning within $p_{f}$ are identified by removing uncontaminated segments at both ends, leaving only contaminated segments that need cleaning (line 12). If segments that need to be cleaned are identified, a globally aware wash operation is performed (lines 13–14). To ensure biochemical integrity, all reaction operations are spatially and temporally isolated from washing tasks. Reactions are enclosed within fixed components, and during cleaning, routing paths are selected to avoid any overlap with sample transportation channels. Therefore, the washing process has no effect on analyte concentration or reaction outcomes.
**Algorithm 1** Path-driven application mapping iteration algorithm**Require:** Binding and scheduling results, chip layout**Ensure:** Application mapping result 1:  flag ← true, $T_{min}\leftarrow \infty$, Q←∅, $Q^{\prime }\leftarrow \emptyset$; 2: **while** flag **do** 3:      Add operations without parent operations to ready queue *Q*; 4:      **while** Q≠∅ or $Q^{\prime }\neq \emptyset$ **do** 5:           **if** $Q^{\prime }\neq \emptyset$ **then** 6:                Call operation sorting algorithm to sort operations in *Q*, and add them to $Q^{\prime }$; 7:           **end if** 8:           Retrieve an operation $o_{i}$ from the front of $Q^{\prime }$; 9:           **if** there are other fluid in the target component *m* **then**10:                Transport the fluid to the next component or a dedicated storage unit;11:           **end if**12:           Calculate the contaminated channel segments in the current flow path;13:           **if** there is a path that needs to be cleaned **then**14:                Perform globally aware wash operation;15:           **end if**16:           Execute transportation task and update component and channel information;17:           Execute $o_{i}$, add $o_{i}$’s child operations to queue *Q*, update component information;18:      **end while**19:      **if** completion time remains unchanged for three consecutive iterations **then**20:           flag ← false;21:      **end if**22:      $T_{min}\leftarrow \min\left(T_{min},T\right)$;23: **end while**

Before executing a fluid transportation task, the start and finish times must be determined, followed by task execution (line 16). Consider a transportation task moving fluid from a source (sour) to a destination (des) along a defined path ($p_{f}$). The source can be a port, component, or storage unit. When sour is an input port, fluid enters the biochip, and the destination must be a component. If sour is a component, the destination can be another component, an output port, or a storage unit. If sour is storage, the destination must be a component. The duration required for fluid transportation from sour to des, denoted $t_{tr}\left(sour,des\right)$, can be calculated as

(19) $$t_{tr}\left(sour,des\right)=t_{prep}+\frac{len(p_{f})}{v_{tr}}$$

where $t_{prep}$ is the valve switching preparation time, $len(p_{f})$ is the length of the path, and $v_{tr}$ is the flow rate of fluids. The start and finish times, $t_{start}\left(sour,des\right)$ and $t_{finish}\left(sour,des\right)$, are calculated as follows:

(20) $$t_{start}\left(sour,des\right)=\max\left(t_{ready}\left(sour\right),t_{ready}\left(des\right),t_{ready}\left(p_{f}\right)\right)$$

(21) $$t_{finish}\left(sour,des\right)=t_{start}\left(sour,des\right)+t_{tr}\left(sour,des\right)$$

where $t_{ready}\left(sour\right)$, $t_{ready}\left(des\right)$, and $t_{ready}\left(p_{f}\right)$ represent the readiness times of the source, destination, and transportation path, respectively, corresponding to the latest completion time of their previous tasks.

After moving fluid from one component *m* to another chip location, ensure that both the transportation path and component *m* are uncontaminated. If contamination is present, the contaminated channels and components are recorded, and a wash operation is initiated. Once component *m* is confirmed free from unnecessary fluid and contamination, the transportation task to move the required fluid to component *m* is executed, followed by operation execution. The start time $t_{start}\left(o_{j}\right)$ and finish time $t_{finish}\left(o_{j}\right)$ for operation execution are calculated as

(22) $$t_{start}\left(o_{j}\right)=\max\left(t_{end}\left(tr\left(e_{k,j}\right)\right)\right),e_{k,j}\in E$$

where $tr(e_{k,j})$ denotes the fluid transportation task delivering output fluid from operation $o_{k}$ to the bound component of operation $o_{j}$.

(23) $$t_{finish}\left(o_{j}\right)=t_{start}\left(o_{j}\right)+t_{e}\left(o_{j}\right)$$

where $t_{e}\left(o_{j}\right)$ is the time required to execute operation $o_{j}$.

Conflicts between fluid transportation and wash operations at intersections or shared channels can cause delays. The delay time $t_{delay}\left(task\right)$ for any task (transportation or cleaning) is defined as

(24) $$t_{delay}\left(task\right)=t_{start}\left(task\right)-t_{ready}\left(task\right)$$

where $t_{start}\left(task\right)$ is the actual start time of the task, and $t_{ready}\left(task\right)$ is its readiness time. The cumulative delay time for all tasks, caused by conflicts or wash operations, serves as the performance evaluation metric for the algorithm presented in this section.

## 5. Experimental Results

The proposed algorithm was implemented in C++ language on a PC with 2.10 GHz CPU and 16 GB memory. Eight bioassays were used to verify the proposed method, as shown in Table 2, in which PCR (Polymerase Chain Reaction) and IVD (In Vitro Diagnostics) are real-life biochemical applications and the other four bioassays are synthetic benchmarks [16]. Moreover, Table 2 shows the details of each benchmark, including |*O*| (the number of biochemical operations), #CP (the number of connection pairs), and the number of components represented in the following format: (ports, mixers, heaters, filters, detectors). The column “Grid” lists the initial sizes of grids for physical design (10 mm/grid unit), that is, the maximum allowed sizes of biochips. Moreover, the other parameters used in this paper are set as follows: $\omega_{start}$ = 0.9, $\omega_{end}$ = 0.2, $h_{1}$ = 0.6, $h_{start}$ = 0.3, $h_{end}$ = 0.8, iterators = 500, $t_{prep}$ = 0.1, and $v_{tr}$ = 10 mm/s.

### 5.1. Validation of Fluid Routing

This study systematically compares the proposed HPSO-CAR routing method, based on discrete particle swarm optimization, with the OAXSMT-DPSO method from reference [32]. By integrating GA and OED into the particle swarm optimization framework, HPSO-CAR significantly enhances search capabilities. The experiments evaluate multiple benchmark microfluidic chip design cases, focusing on three critical performance metrics: the number of intersections, total channel length, and the total congestion time. The experimental results are shown in Table 3.

In terms of intersection reduction, the proposed method achieved improvements in five out of eight test cases, with reductions ranging from 3.13% to 20.00%, and an average improvement of 8.15%. Notably, intersection reductions of 20.00%, 16.67%, and 14.29% were achieved in PCR, IVD-3, and sy20 test cases, respectively. Channel length optimization was accomplished in all test cases, with improvements ranging from 6.37% to 18.50%, averaging 13.21%. Specifically, the PCR and IVD-1 test cases showed remarkable reductions of 18.50% and 17.62%, respectively, demonstrating significant advantages of the proposed algorithm in microfluidic routing. Regarding fluid transport conflict time, the proposed method reduced congestion times in six test cases, with improvement rates ranging from 4.76% to 10.53%, averaging a reduction of 5.32%. Particularly noteworthy are the IVD-2 and sy15 cases, which exhibited improvements of 10.00% and 10.53%, respectively.

This study compares our proposed HPSO-CAR-based routing method with the A*-based routing approach presented in reference [13]. Although [13] incorporates washing costs and routing conflicts within its algorithm, its optimization results are limited due to the heuristic-based single-pass routing strategy employed. In contrast, our HPSO-CAR algorithm significantly enhances global search capabilities and local optimization efficiency by organically integrating GA and OED into a DPSO framework. The experimental results are shown in Table 4.

The HPSO-CAR algorithm demonstrated exceptional performance in reducing the number of intersections, achieving significant reductions in seven out of eight test cases, with an average improvement rate of 21.79%. Particularly noteworthy are the PCR and sy20 test cases, where intersection counts were reduced by 33.33% and 35.71%, respectively. These results clearly indicate the algorithm’s prominent advantage in lowering the topological complexity of microfluidic chips. Regarding total channel length, the HPSO-CAR algorithm achieved significant optimization across all test cases, averaging a reduction of 22.05%. In the PCR test case, the channel length decreased by 37.20% and, in the IVD-2 case, by 28.11%. These outcomes demonstrate the algorithm’s superior capability for efficiently planning microfluidic channel layouts, significantly reducing the required chip area and consequently lowering manufacturing costs. In terms of conflict time for fluid transportation tasks, all test cases except PCR exhibited varying degrees of improvement, with an average optimization rate of 11.93%. The sy15 showed a notable congestion-time reduction of 29.17%, and the IVD-3 reduced congestion time by 17.39%, clearly underscoring the algorithm’s advantage in enhancing parallelism and the temporal efficiency of microfluidic operations.

These experimental results strongly validate the effectiveness of integrating multiple optimization strategies in our proposed approach. By combining the diverse exploration mechanisms of GA, the precise local optimization properties of OED, and the powerful global search capability of PSO, the HPSO-CAR algorithm achieves a balanced approach between global optimization and fine-grained local adjustment, leading to significant performance improvements in the physical synthesis of microfluidic chips.

### 5.2. Validation of Application Mapping

The comparative experiments between the proposed path-driven application mapping algorithm and the traditional LSAM-NR (list-scheduling-based application mapping—naive rinsing) method [16] demonstrate significant performance improvements. LSAM-NR does not allow other fluidic-handling operations to be performed in parallel during the wash operation, which makes the delay time increase substantially and leads to a large increase in the biochemical reaction completion time. The experimental results are shown in Table 5. Regarding total delay time, our algorithm achieved an average reduction of 36.33% across eight test cases. Notably, the improvement is attributed to intelligently optimized fluid routing, which effectively resolves the delay problems encountered by the LSTM-NR algorithm during washing steps, such as in the sy20 and sy10 test cases, highlighting the robustness of our approach. In terms of total biochemical reaction execution time, our algorithm demonstrated excellent performance with an average optimization rate of 18.76%. The overall trend analysis shows that algorithm performance improvements are positively correlated with the complexity of the test cases, demonstrating that our approach is particularly suitable for solving complex microfluidic scheduling problems. The algorithm also exhibits good adaptability and robustness, with no extreme differences observed in optimization across different metrics, such as delay time or execution time.

This study’s comparative experiments between the proposed path-driven application mapping algorithm and the LSTM-RINS (list scheduling-based application mapping—rinsing integrated scheduling) approach [16] demonstrate significant performance improvements. When executing wash operations, the LSAM-RINS algorithm can simultaneously perform fluid transportation tasks and bioassay operations, but it only conducts scheduling once. When conflicts occur between fluid transportation tasks, it can only delay one of the tasks without making adjustments. Additionally, the wash operation targets as many multi-polluted channels and components as possible without considering whether these channels and components will be used again. This increases unnecessary cleaning, delays the execution time of operations and fluid transportation tasks, increases delay time, and consequently extends the completion time of biochemical reactions. The experimental results are shown in Table 6.

In terms of total delay time, our algorithm achieved an average delay reduction of 21.97% across all eight test cases. The sy10 test case notably stood out with a 35.19% reduction in delay time. These improvements primarily stem from intelligently optimizing operational sequencing through iterative adjustments and effectively resolving fluid transportation conflicts, overcoming delay issues arising from the simplistic washing strategy employed by the LSTM-RINS method. Regarding total biochemical reaction execution time, the algorithm achieved an average reduction of 8.30%. Although this reduction is relatively smaller than the delay time improvements, it still highlights a significant enhancement in overall microfluidic operational efficiency. In particular, in sy10 and IVD-2, execution times were reduced by 16.67% and 13.31%, respectively. The variation in improvement among cases may be attributed to specific structures and operational dependencies within each test scenario.

Further analysis indicates that the advantages of our algorithm over the LSTM-RINS approach primarily manifest in two key aspects: firstly, conflicts between fluid routing tasks were more intelligently resolved through multi-iteration adjustments of operational sequences; secondly, wash operations were precisely managed to avoid unnecessary delays. Overall, these experimental results clearly validate the effectiveness of the proposed path-driven application mapping algorithm for optimizing microfluidic biochip applications. By intelligently adjusting operation sequences and precisely managing washing tasks, the algorithm successfully overcomes the limitations inherent in the LSTM-RINS method, significantly improving microfluidic chip execution performance.

## 6. Future Prospects

Future Directions: While the proposed framework focuses on system-level routing and scheduling optimization for continuous-flow microfluidic biochips, it currently does not consider detailed fluidic properties such as interfacial tension, contact angle, viscosity (Newtonian or non-Newtonian behavior), or channel surface material. These properties can significantly impact transportation speed and cleaning efficiency, especially for highly viscous or non-Newtonian fluids. To address the limitation concerning the modeling of fluid rheology and viscosity, future improvements should incorporate advanced computational fluid dynamics (CFD) models explicitly designed for non-Newtonian fluids. Specifically, integrating rheological models such as power-law or Carreau–Yasuda models into the fluid transport and wash operation calculations would better reflect realistic fluid behavior. Additionally, empirical data from microfluidic experiments involving common biochemical reagents (e.g., blood samples, emulsions) can be employed to calibrate and validate these advanced models, thereby increasing the accuracy and reliability of the proposed scheduling framework. Moreover, the robustness of our methodology under higher flow rates deserves further investigation. Our approach currently assumes laminar flow regimes, maintaining accuracy for flow rates up to approximately 10 mm/s in typical microchannels. Beyond this threshold, inertial effects become significant, requiring modified delay time calculations and specialized component models. For applications needing enhanced mixing or high throughput where transitional flow may occur, appropriate extensions to our framework could support these specialized requirements.

Application Scenarios: The proposed path-driven fluid routing and scheduling methodology holds significant potential in several practical application domains. In clinical diagnostics, where rapid and accurate sample processing is critical, our approach can facilitate low-latency biochemical workflows for point-of-care testing, such as infectious disease screening or cancer biomarker detection. By minimizing transportation delays and wash overheads, the method enhances assay throughput and reliability under time-sensitive constraints. In pharmaceutical development, especially in high-throughput drug screening and microreactor-based synthesis, the ability to tightly coordinate complex sequences of biochemical operations is essential. Our framework’s conflict-aware scheduling and efficient channel reuse can support scalable assay pipelines, reducing cross-contamination and resource consumption. In environmental monitoring, continuous-flow microfluidic systems are increasingly used for the real-time detection of pollutants and toxins in field conditions. The delay-optimized design flow can improve responsiveness and operational robustness in such distributed, autonomous platforms, where reliability and minimal reagent usage are paramount.

Practical Implementation: To bridge the gap between theoretical advancements and practical utilization, several key implementation strategies are recommended. Our proposed path-driven method can be effectively integrated into widely used CAD software such as AutoCAD 2025 or SolidWorks 2024 via existing Application Programming Interfaces (APIs), facilitating automated and streamlined design processes. Leveraging parallel computation methods such as GPU acceleration or multi-threading available in contemporary CAD systems can significantly enhance computational efficiency, enabling faster and more interactive design iterations. Additionally, we emphasize targeted application scenarios, including clinical diagnostics, pharmaceutical drug screening, and environmental monitoring, where our method notably improves assay performance and reduces contamination risks. Finally, developing intuitive graphical user interfaces (GUIs) to visually specify design constraints and interactively refine routing and scheduling outcomes is essential to encourage broader adoption and usability among biochip designers and practitioners.

## 7. Conclusions

This paper presents an integrated path-driven methodology that jointly optimizes routing and application mapping for continuous-flow microfluidic biochips. For routing, we developed a conflict-aware algorithm based on hybrid particle swarm optimization that balances channel length minimization, crossing point reduction, and conflict avoidance. For application mapping, we proposed an iterative scheduling approach that leverages historical information to progressively optimize both transport and washing operations, overcoming limitations of traditional one-shot scheduling. Comprehensive experiments demonstrated significant improvements over state-of-the-art methods: 22.05% reduction in channel length, 21.79% fewer crossing points, 21.97% lower delay time, and 8.30% shorter biochemical reaction completion time. These results validate the benefits of our integrated approach considering both physical and operational aspects in CFMB design.

## Figures and Tables

**Table 1:** Fluid transportation tasks.

Connection Pair	Transportation Task	Time Interval (s)
$Con_{1}$	a→b	(1, 3), (12, 14)
$Con_{2}$	b→c	(0, 2), (4, 6), (6, 8), (8, 10)
$Con_{3}$	a→d	(4, 6), (6, 8), (8, 10)
$Con_{4}$	c→d	(14, 16)

**Table 2:** Details of benchmarks used in the experiments.

Benchmark	|*O*|	Grid	#CP	Number of Components
IVD-1	12	36 × 36	10	(4, 2, 0, 0, 2)
IVD-2	18	54 × 54	15	(6, 3, 0, 0, 3)
IVD-3	24	54 × 54	15	(6, 3, 0, 0, 3)
PCR	7	27 × 27	9	(3, 3, 0, 0, 0)
sy10	10	54 × 54	18	(5, 2, 1, 1, 1)
sy15	15	72 × 72	30	(8, 3, 2, 1, 2)
sy20	20	81 × 81	31	(9, 3, 3, 2, 1)
sy25	25	81 × 81	33	(8, 4, 2, 1, 1)

**Table 3:** Comparison of the proposed routing method and the OAXSMT-DPSO method in [32].

Benchmark	Number of Intersections			Total Channel Length (mm)			Total Congestion Time (s)		
	[32]	Ours	Imp (%)	[32]	Ours	Imp (%)	[32]	Ours	Imp (%)
IVD-1	5	5	0.00	615.7	507.2	17.62	6	6	0.00
IVD-2	4	4	0.00	827.6	694.1	16.13	20	18	10.00
IVD-3	6	5	16.67	1754.9	1643.1	6.37	20	19	5.00
PCR	5	4	20.00	653.5	532.6	18.50	4	4	0.00
sy10	5	5	0.00	981.9	845.3	13.91	21	20	4.76
sy15	18	16	11.11	2644.7	2424.8	8.31	19	17	10.53
sy20	21	18	14.29	2532.3	2172.8	14.20	32	29	9.38
sy25	32	31	3.13	2963.4	2648.9	10.61	37	34	8.11
Average	-	-	8.15	-	-	13.21	-	-	5.32

**Table 4:** Comparison of the proposed routing method and A*-based routing method in [13].

Benchmark	Number of Intersections			Total Channel Length (mm)			Total Congestion Time (s)		
	[13]	Ours	Imp (%)	[13]	Ours	Imp (%)	[13]	Ours	Imp (%)
IVD-1	5	5	0.00	631.3	507.2	19.66	7	6	14.29
IVD-2	5	4	20.00	965.5	694.1	28.11	19	18	5.26
IVD-3	6	5	16.67	1980.2	1643.1	17.02	23	19	17.39
PCR	6	4	33.33	848.1	532.6	37.20	4	4	0.00
sy10	7	5	28.57	1105.9	845.3	23.56	22	20	9.09
sy15	21	16	23.81	2745.7	2424.8	11.69	24	17	29.17
sy20	28	18	35.71	2869.3	2172.8	24.27	34	29	14.71
sy25	37	31	16.22	3113.4	2648.9	14.92	36	34	5.56
Average	-	-	21.79	-	-	22.05	-	-	11.93

**Table 5:** Comparison of experimental results with LSAM-NR.

Benchmark	Total Delay Time (s)			Total Execution Time (s)		
	LSAM-NR	Ours	Imp (%)	LSAM-NR	Ours	Imp (%)
IVD-1	30.3	18.4	39.27	68.4	58.4	17.12
IVD-2	59.2	44.1	25.51	84.6	74.4	13.71
IVD-3	124.7	72.4	41.94	216.9	177.5	22.23
PCR	5.5	4.3	21.82	39.9	39.1	2.12
sy10	66.9	37.2	44.39	137.8	111.1	24.05
sy15	96.4	65.3	32.26	184.1	158.0	16.53
sy20	104.6	52.5	49.81	221.2	165.1	34.02
sy25	194.6	125.3	35.61	362.4	301.3	20.29
Average	-	-	36.33	-	-	18.76

**Table 6:** Comparison of experimental results with LSTM-RINS.

Benchmark	Total Delay Time (s)			Total Execution Time (s)		
	LSTM-RINS	Ours	Imp (%)	LSTM-RINS	Ours	Imp (%)
IVD-1	22.7	18.4	18.94	62.7	58.4	7.36
IVD-2	56.2	44.1	21.53	84.3	74.4	13.31
IVD-3	93.5	72.4	22.57	194.9	177.5	9.83
PCR	5.5	4.3	21.82	39.9	39.1	2.12
sy10	57.4	37.2	35.19	129.6	111.1	16.67
sy15	77.1	65.3	15.30	161.4	158.0	2.15
sy20	73.3	52.5	28.38	184.1	165.1	11.50
sy25	142.4	125.3	12.01	311.6	301.3	3.41
Average	-	-	21.97	-	-	8.30

## Data Availability

The data presented in this study are available on request from the corresponding author.

## Nomenclature

The following Nomenclatures are used in this manuscript:

**Symbol**	**Description**
ω	Inertia weight for velocity update
*c*	Acceleration constant
*r*	Random number in [0,1]
$P_{o}$	Guide particle constructed using OED from $P_{b}$ and $G_{b}$
$P_{b}$	Historical best position of the particle
$G_{b}$	Global best position found by the swarm
$h_{1},\;h_{2}$	Probabilities for mutation and crossover operations
$X_{i}^{t}$	Position of particle *i* at iteration *t*
$V_{i}^{t}$	Velocity of particle *i* at iteration *t*
$F_{1},\;F_{2},\;F_{3}$	Functions for mutation, OED, and crossover
L[a][b]	Element of orthogonal array at row *a*, column *b*
*M*	Number of experimental particles or rows in the array
*D*	Number of dimensions (equal to number of connection pairs)
*u*	$\log_{2}M$, used for orthogonal array construction
$k_{ijq}$	0–1 indicator in orthogonal matrix
$S_{jq}$	Score of level *q* in dimension *j*
CN	Number of intersections in routing
*L*	Total length of channels
$T_{\mathrm{total}}$	Total conflict time among connection pairs
$\mathrm{Fitness}(X_{i})$	Fitness value of particle *i*
it	Current iteration number
iterators	Maximum number of iterations
$\omega_{\mathrm{start}},\;\omega_{\mathrm{end}}$	Start/end values of inertia weight
$h_{\mathrm{start}},\;h_{\mathrm{end}}$	Start/end values of crossover probability
